# System of Night Practice in Paris

**Published:** 1876-08

**Authors:** 


System of Night Practice in Paris.—The plan on which night practice is arranged in Paris seems equally convenient for the patient and practitioner. The Municipal Council have caused lists of medical men willing to be called to patients in the night to be suspended in all the police stations. When assistance is required, the friends repair to the office, choose a medical man from the list, and are taken to his residence by a police officer, who also accompanies the doctor to the patient's house. For this visit ten francs are to be paid, either by the sick person or the authorities, according to the patient's circumstances.—*Med. and Surg. Reporter.*

